# Improving the prediction for the response to radiotherapy of clinical tumor samples by using combinatorial model of MicroRNA expression

**DOI:** 10.3389/fgene.2022.1069112

**Published:** 2022-11-22

**Authors:** Chao Tang, Jun Qi, Yan Wu, Ling Luo, Ying Wang, Yongzhong Wu, Xiaolong Shi

**Affiliations:** Radiation and Cancer Biology Laboratory, Radiation Oncology Center, Chongqing Key Laboratory of Translational Research for Cancer Metastasis and Individualized Treatment, Chongqing Cancer Hospital, Chongqing University Cancer Hospital and Chongqing Cancer Institution, Chongqing, China

**Keywords:** radiotherapy, radioresistance, miRNA expression, combinatorial model, TCGA databases, overall survival

## Abstract

**Purpose:** Radiation therapy (RT) is one of the main treatments for cancer. The response to radiotherapy varies widely between individuals and some patients have poor response to RT treatment due to tumor radioresistance. Stratifying patients according to molecular signatures of individual tumor characteristics can improve clinical treatment. In here, we aimed to use clinical and genomic databases to develop miRNA signatures that can predict response to radiotherapy in various cancer types.

**Methods:** We analyzed the miRNAs profiles using tumor samples treated with RT across eight types of human cancers from TCGA database. These samples were divided into response group (S, *n* = 224) and progressive disease group (R, *n* = 134) based on RT response of tumors. To enhance the discrimination for S and R samples, the predictive models based on binary logistic regression were developed to identify the best combinations of multiple miRNAs.

**Results:** The miRNAs differentially expressed between the groups S and R in each caner type were identified. Total 47 miRNAs were identified in eight cancer types (*p* values <0.05, t-test), including several miRNAs previously reported to be associated with radiotherapy sensitivity. Functional enrichment analysis revealed that epithelial-to-mesenchymal transition (EMT), stem cell, NF-κB signal, immune response, cell death, cell cycle, and DNA damage response and DNA damage repair processes were significantly enriched. The cancer-type-specific miRNA signatures were identified, which consist of 2-13 of miRNAs in each caner type. Receiver operating characteristic (ROC) analyses showed that the most of individual miRNAs were effective in distinguishing responsive and non-responsive patients (the area under the curve (AUC) ranging from 0.606 to 0.889). The patient stratification was further improved by applying the combinatorial model of miRNA expression (AUC ranging from 0.711 to 0.992). Also, five miRNAs that were significantly associated with overall survival were identified as prognostic miRNAs.

**Conclusion:** These mRNA signatures could be used as potential biomarkers selecting patients who will benefit from radiotherapy. Our study identified a series of miRNA that were differentially expressed between RT good responders and poor responders, providing useful clues for further functional assays to demonstrate a possible regulatory role in radioresistance.

## 1 Introduction

Radiotherapy (RT) plays a crucial role in cancer treatment and more than half of all cancer patients receive RT during the course of disease (Baskar et al., 2012). In last 20 years, the outcomes of RT have been improved dramatically due to the developments of highly conformal RT techniques, such as intensity-modulated RT (IMRT), intensity-modulated arc therapy (IMAT) and stereotactic RT (SRT) (Minniti et al., 2021). However, unfortunately the outcomes of therapy are not fully satisfactory. The response to radiotherapy varies widely between individuals and some patients are resistant to RT treatment. Radioresistance is considered as a main factor impeding efficacy of radiotherapy. Although radioresistance has been implicated to be associated with several biological alterations of the tumor cells, such as tumor metabolism (Tang et al., 2018), cell cycle arrest (Chen et al., 2017), oncogene and tumor suppressor alterations (Pitroda et al., 2009), microenvironment (TME) change (Suwa et al., 2021), autophagic regulation (Chang et al., 2014), cancer stem cells (CSCs) generation (Ning et al., 2013), and DNA damage response (DDR) and repair (Huang and Zhou, 2020; Sun et al., 2020), the mechanisms underlying resistance to radiation are still largely obscure. Therefore, uncovering the processes and charactering the molecules associated with regulation of radioresistance may lead to improved, efficient treatment for cancer patients.

Previous studies revealed that the difference in the status of mutation and expression profile of gene including microRNAs (miRNAs), is associated with radioresistance (Mercatelli et al., 2008; Huang et al., 2013; Mueller et al., 2013; Hatano et al., 2015; Mao et al., 2016; Xu et al., 2016; Tao et al., 2018; Xu et al., 2018). MicroRNAs are small non-coding single-stranded RNAs of 19–23 nucleotides in length, which play a critical role in post-transcriptional regulation by degrading or preventing the translation of their target messenger RNA (mRNA). They play important roles in tumor development and metastasis. Emerging evidences demonstrated that miRNAs may be involved in regulation processes associated with response to radiation. For examples, previous studies revealed that some microRNAs, such as miR-95 (Huang et al., 2013), miR-221, miR-222 (Mercatelli et al., 2008), and miR-106b (Hatano et al., 2015) enhanced radioresistance in cancer cells, while miR-30a (Xu et al., 2016), miR-16 (Tao et al., 2018), miR-449 (Mao et al., 2016), miR-17 (Xu et al., 2018), and miR-100 (Mueller et al., 2013) enhanced the radiosensitivity. Several miRNAs regulate DNA damage response (DDR) pathway. For example, miR-101 can regulate the expression of ataxia-telangiectasia mutated (ATM) gene and DNA-PK. ATM is a central regulator of DNA damage response (Maréchal and Zou, 2013). Mutation and inactivation of ATM can lead to increased instability of genome and impaired repair ability for DNA double-strand breaks. It has been experimentally demonstrated that up-regulation of miR-101 reduced the protein levels of DNA-PK and ATM, rendering tumor cells more sensitive to radiation (Yan et al., 2010). MiR-223 is a regulator of maturation and differentiation of hematopoietic stem cells. Up-regulation of miR-223 will reduce the expression of ATM and make U87 cells sensitive to radiation *in vitro* (Liang et al., 2014). MiR-375 is a negative regulator of p53 which is a key tumor suppressor gene that inhibits cell growth by activating cell cycle arrest or apoptosis (Vousden and Prives, 2009). Increase of miR-375 was detected in recurrent gastric cancer (Zhang et al., 2011), and the increased miR-375 interacts with the 3′UTR of p53 gene that negatively regulates p53 and its downstream pathway genes, resulting in radioresistance of cells to radiation (Liu et al., 2013).

In the era of precision medicine, a biomarker of intrinsic radiosensitivity would be extremely valuable for selecting patients in whom will benefit from RT, adjusting individual dosing, and aiding decision making. Previous studies have demonstrated that several miRNAs were associated with radioresistance or radiosensitivity, implying they could serve as promising biomarkers for prediction of RT response. Besides, miRNAs serving as biomarkers have several advantages. Firstly, unlike mRNAs, miRNAs remain largely intact in routinely collected, formalin-fixed, paraffin-embedded (FFPE) clinical tissues. Therefore, the detection for miRNA levels could be conveniently performed in clinical practice. Secondly, miRNAs have been called ‘the master regulators’ of gene expression since a single miRNA can regulate several hundreds of mRNA targets. Researches have indicated that many cell phenotype or subtype is likely governed by a miRNA regulatory network (Yang D. et al., 2013), suggesting some miRNAs may sever as the possible major determinants of cellular phenotype (including radioresistant phenotype) (McDermott et al., 2017). In additional, cell lines are usually used as models for radioresistance research in previous reports. However, tumors from patients are heterogeneous. Thus, the factors related to RT response are more complex than in cell lines. On the other hand, the results from cellular experiments also need to be confirmed with clinical samples. Therefore, we proposed that miRNA profile analysis based on clinical samples could provide a direct assessment for their performances as biomarkers for prediction of RT response. In this study, we performed a large-scale analysis of miRNA expression data collected from The Cancer Genome Atlas (TCGA) from those who treated with RT across eight cancer types. By analyzing the expressional difference between RT response and progressive disease samples, the miRNA signatures for predicting RT response were obtained and their performance for prediction of RT response were estimated.

## 2 Materials and methods

### 2.1 Data collection

MiRNA expression data of cancer patients undergoing RT were downloaded from the website UCSC XENA - GDC TCGA (https://xenabrowser.net/hub/). The expression profiles were presented as RPM (reads of exon model per million mapped reads). Clinical information including patient’s response to RT and overall survival was downloaded from GDC TCGA website (https://portal.gdc.cancer.gov/). The tumor samples were categorized into complete response (S) and progressive disease (R) groups depending on their clinical response to RT treatment. Complete response and progressive disease was defined according to RECIST. Finally, we analyzed 358 clinical samples across eight different cancer types, including S group (*n* = 224) and R group (*n* = 134).

### 2.2 Differential expression analysis

The differential expression analysis of miRNAs was performed in each cancer type. The miRNA would not be analyzed further if the reads of these miRNAs were empty in more than 10% of the samples. Deseq2 package was used to normalize miRNA data and identify the difference of miRNA expression levels between S and R group (Love et al., 2014). Wald test and t-test was used to calculate the *p*-value. In this study, *p* < 0.05 and | logfc | > 1 were used as threshold criteria for screening DEMs between S and R group.

### 2.3 Identification of DEMs

The identification of differentially expressed miRNA (DEMs) was conducted using R language. The expression levels of DEMs were visualized using the ggpubr, ggplot2 and complexheatmap packages. The receiver operating characteristic (ROC) curve was drawn and the area under the curve (AUC) was calculated using the pROC package. Heatmap of miRNA expression levels for individual cancer types was drawn using pheatmap. Kaplan Meier curve of single gene was drawn by Survminer package. The samples were divided into high expression group and low expression group according to the median expression level of miRNA and *p*-value was calculated by log-rank test. Enrichment analysis of the 47 DEMs was performed by the online tool TAM 2.0 (http://www.lirmed.com/tam2/).

### 2.4 Combinatorial models of multiple miRNAs

A logistic regression model was developed by combining expressions of multiple miRNAs in each cancer type. In our study, the dependent variable of this model was the normalized expression level of miRNA; the independent variable was the RT response of samples (response or progressive disease). The method of combinatorial modelling was described in detail (Xu et al., 2022). The samples were randomly divided into a training set and a test set. The training set contained 80% of the total samples, and the test set contained 20% of the total samples. The *K*-fold cross-validation (*K* = 5) was used to fit the combinatorial model. We used different combinations of the data groups that were partitioned to train and test *K* different models, and then the performance was evaluated. In running the final model, we found that the independent variable were the most accurate predictors of the dependent variable. The formula of logistic regression is as follows:

Y=W1 * miRNA1+W2 * miRNA2+W3 * miRNA3+……+Wn * miRNAn+intercept.

*Y* is the predictive index of RT response. The optimal threshold for dividing S and R groups in the training set was calculated, and then the test set was tested using the threshold. If the predictive index is more than the threshold, it indicates that the sample is more likely to be radiosensitive; otherwise, the sample is more likely to be radioresistant. Finally, the area under the receiver operating characteristic curve was calculated to evaluate the predictive ability.

## 3 Results

### 3.1 Clinical information of cancer patients

**FIGURE 1:**
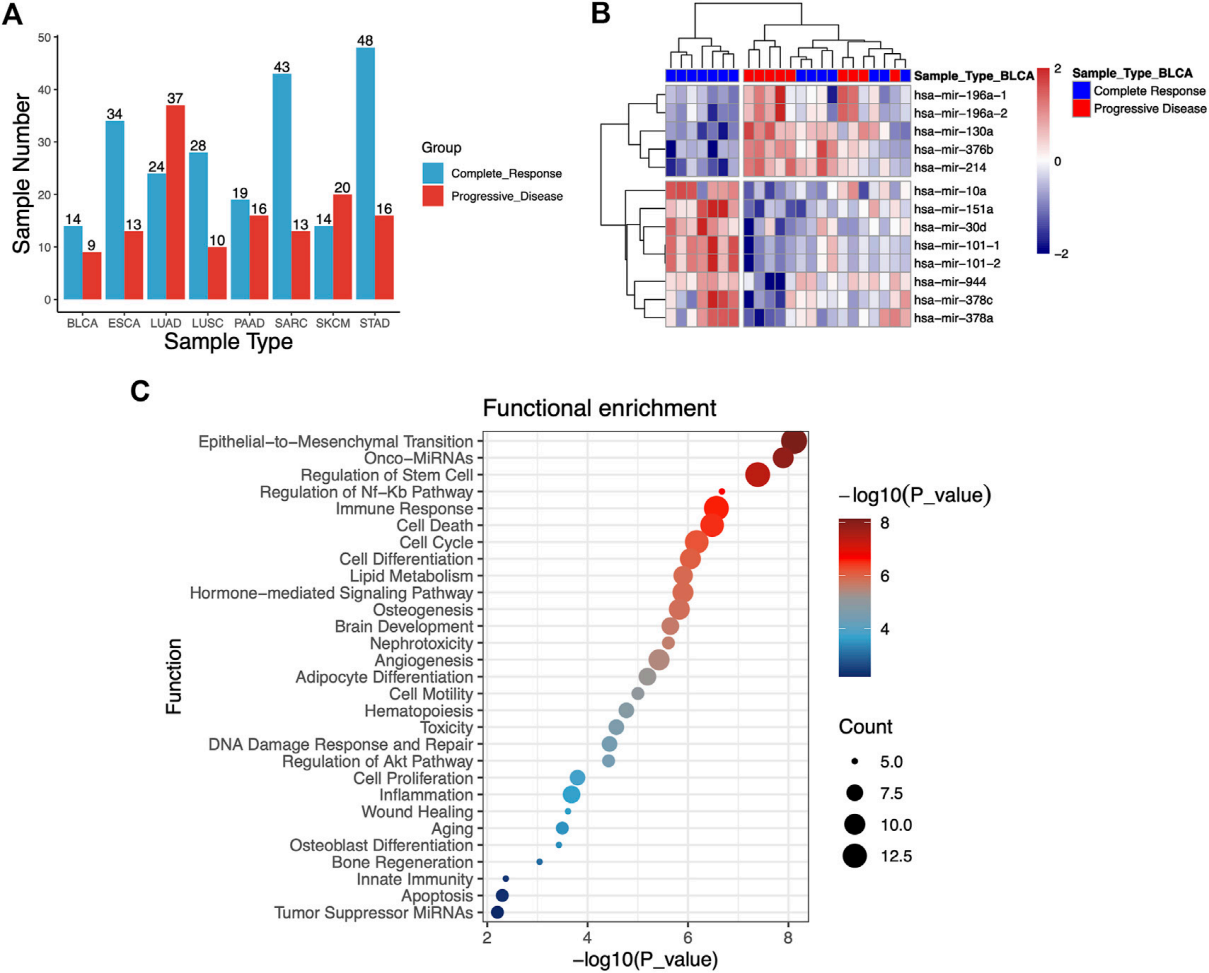
**(A)** Statistical histogram of cancer types of samples and grouping. The samples of cancer patients were categorized into complete response (S) group and progressive disease (R) group depending on their clinical response to RT treatment. A total of 358 tumor samples across eight different types of human cancers from TCGA database are analyzed. **(B)** Heatmap of 13 miRNAs that were differentially expressed from S and R samples in the bladder urothelial carcinoma (BLCA). **(C)** Functional enrichment analysis of 47 differentially expressed miRNAs.

To investigate the association between RT response and miRNA profiles, we analyzed miRNA expression data of patients undergoing RT from TCGA database. A total of 358 clinical samples across eight different types of human cancers were analyzed in this study, including bladder urothelial carcinoma (BLCA, *n* = 23), esophageal carcinoma (ESCA, *n* = 47), lung adenocarcinoma (LUAD, *n* = 61), lung squamous cell carcinoma (LUSC, *n* = 38), pancreatic cancer (PAAD, *n* = 35), sarcoma (SARC, *n* = 56), skin cutaneous melanoma (SKCM, *n* = 34) and stomach adenocarcinoma (STAD, *n* = 64). The samples of cancer patients were categorized into complete response (S) group and progressive disease (R) group depending on their clinical response to RT treatment. Finally, 358 clinical samples were analyzed, including S group (*n* = 224) and R group (*n* = 134). The cancer types and groups of samples were shown in Figure 1A.

### 3.2 Identification of DEMs in group S and R

To identify potential miRNA biomarkers, it is essential to compare miRNAs differentially expressed from RT response samples (S) and disease progression samples (R). According to the screening criteria of *p*-value <0.05 and | logfc | > 1, the miRNAs differentially expressed (DEMs) between the groups S and R in each caner type were identified. Each cancer type consisted of 2–13 of these miRNAs, and total 47 miRNAs were identified in eight cancer types (Table 1). Hierarchical unsupervised clustering analysis were performed to visualize the expression patterns of these 47 miRNAs. The results showed that these DEMs were cancer type-specific. For example, 13 miRNAs were identified in BLCA, five of them tended to be highly expressed in R group, including miR-196a-1 (*p* = 0.0048), miR-196a-2 (*p* = 0.005), miR-130a (*p* = 0.0163), miR-376b (*p* = 0.0078) and miR-30d (*p* = 0.0016), and eight miRNAs including miR-10a (*p* = 0.021), miR-151a (*p* = 0.013), miR-101-1 (*p* = 0.0007), miR-101-2 ((*p* = 0.0007), miR-944 (*p* = 0.049), miR-378c (*p* = 0.0153), miR-378a (*p* = 0.0158), and miR-214 (*p* = 0.0162) tended to be highly expressed in S (Figure 1B). Likewise, 4-miRNA signature was identified in LUSC. The high expression of miR-937 (*p* = 0.0154) was significantly enriched in R group, while the high expression of miR-592 (*p* = 0.0026), miR-3653 (*p* = 0.0214), miR-628 (*p* = 0.0222) and miR-106a (*p* = 0.0256) was significantly enriched in S group. The details were shown in Table 1.

**TABLE 1:** The differentially expressed miRNAs (DEMs) between RT responsive group (S) and the progressive disease group (R).

MiRNA	Log2FoldChange	*p*-Value	Cancer type	Expression in RT responsive group
miR-101-1	−1.0576	0.0007	BLCA	Up-regulation
miR-101-2	−1.0539	0.0007	BLCA	Up-regulation
miR-30d	−1.5346	0.0016	BLCA	Up-regulation
miR-196a-1	4.3973	0.0048	BLCA	Down-regulation
miR-196a-2	4.5201	0.0050	BLCA	Down-regulation
miR-376b	1.0590	0.0078	BLCA	Down-regulation
miR-151a	−1.2003	0.0130	BLCA	Up-regulation
miR-378c	−1.1853	0.0153	BLCA	Up-regulation
miR-214	1.0717	0.0162	BLCA	Down-regulation
miR-130a	1.3459	0.0163	BLCA	Down-regulation
miR-378a	−1.1258	0.0185	BLCA	Up-regulation
miR-10a	−1.7526	0.0210	BLCA	Up-regulation
miR-944	−1.0781	0.0490	BLCA	Up-regulation
miR-1245a	−1.5099	0.0028	ESCA	Up-regulation
miR-143	−1.4273	0.0045	ESCA	Up-regulation
let-7e	−1.0942	0.0109	ESCA	Up-regulation
miR-142	1.6184	0.0348	ESCA	Down-regulation
miR-194-1	1.6854	0.0022	LUAD	Down-regulation
miR-194-2	1.6576	0.0024	LUAD	Down-regulation
miR-192	1.7344	0.0027	LUAD	Down-regulation
miR-215	1.8478	0.0282	LUAD	Down-regulation
miR-592	−1.5403	0.0026	LUSC	Up-regulation
miR-937	1.2398	0.0154	LUSC	Down-regulation
miR-3653	−1.0457	0.0214	LUSC	Up-regulation
miR-628	−1.4820	0.0222	LUSC	Up-regulation
miR-106a	−1.2683	0.0256	LUSC	Up-regulation
miR-129-2	−1.9278	0.0060	PAAD	Up-regulation
miR-129-1	−1.8086	0.0096	PAAD	Up-regulation
miR-1224	−1.9754	0.0190	PAAD	Up-regulation
miR-29a	−1.1421	0.0057	SARC	Up-regulation
miR-29b-1	−1.3782	0.0097	SARC	Up-regulation
miR-29b-2	−1.3267	0.0112	SARC	Up-regulation
miR-222	−1.2618	0.0200	SARC	Up-regulation
miR-34a	−1.0199	0.0241	SARC	Up-regulation
miR-9-3	1.2433	0.0257	SARC	Down-regulation
miR-9-1	1.2524	0.0258	SARC	Down-regulation
miR-9-2	1.2598	0.0267	SARC	Down-regulation
miR-582	2.1287	0.0276	SARC	Down-regulation
miR-146a	−1.4991	0.0366	SARC	Up-regulation
miR-221	−1.1561	0.0386	SARC	Up-regulation
miR-150	−2.2734	0.0438	SARC	Up-regulation
miR-3917	1.4861	0.0037	SKCM	Down-regulation
miR-33a	1.0720	0.0081	SKCM	Down-regulation
miR-3130-1	1.1210	0.0204	SKCM	Down-regulation
miR-3614	1.2602	0.0360	SKCM	Down-regulation
miR-99a	1.3048	0.0014	STAD	Down-regulation
miR-655	1.0519	0.0085	STAD	Down-regulation

Mapping the differentially expressed miRNAs to signal transduction pathways is important toward understanding their significance in radioresistance. Therefore, we performed enrichment analysis to identify significantly enriched terms. As shown in Figure 1C, the identified pathways were primarily involved in regulation of epithelial-to-mesenchymal transition and stem cell, NF-κB pathway, immune response, cell death and cell cycle. EMT plays a critical role not only in tumor metastasis but also in tumor radioresistance. Epithelial–mesenchymal transition could increase radioresistance (Zhang et al., 2014). We found that EMT signalling pathway to be among the top enriched pathways, implying that the radioresistant phenotype of tumour could be largely explained by the enhancement of EMT pathway. Activation of DNA damage response and DNA damage repair pathway has been demonstrated to be involved in radioresistance (Vousden and Prives, 2009; Yan et al., 2010; Liu et al., 2013; Liang et al., 2014). Enrichment analysis revealed that several DEMs including miR-34a, miR-146a, miR-130a, miR-196a, miR-143, and miR-101 were involved in DNA damage response and DNA damage repair. We also found that several miRNAs (miR-1224, miR-33a, and miR-142) were involved in inflammatory process which was implicated to be a critical radio-response in radioresistant lung cancer cells (Yang H. J. et al., 2013).

### 3.3 Distinguishing S and R group with a single miRNA

**FIGURE 2:**
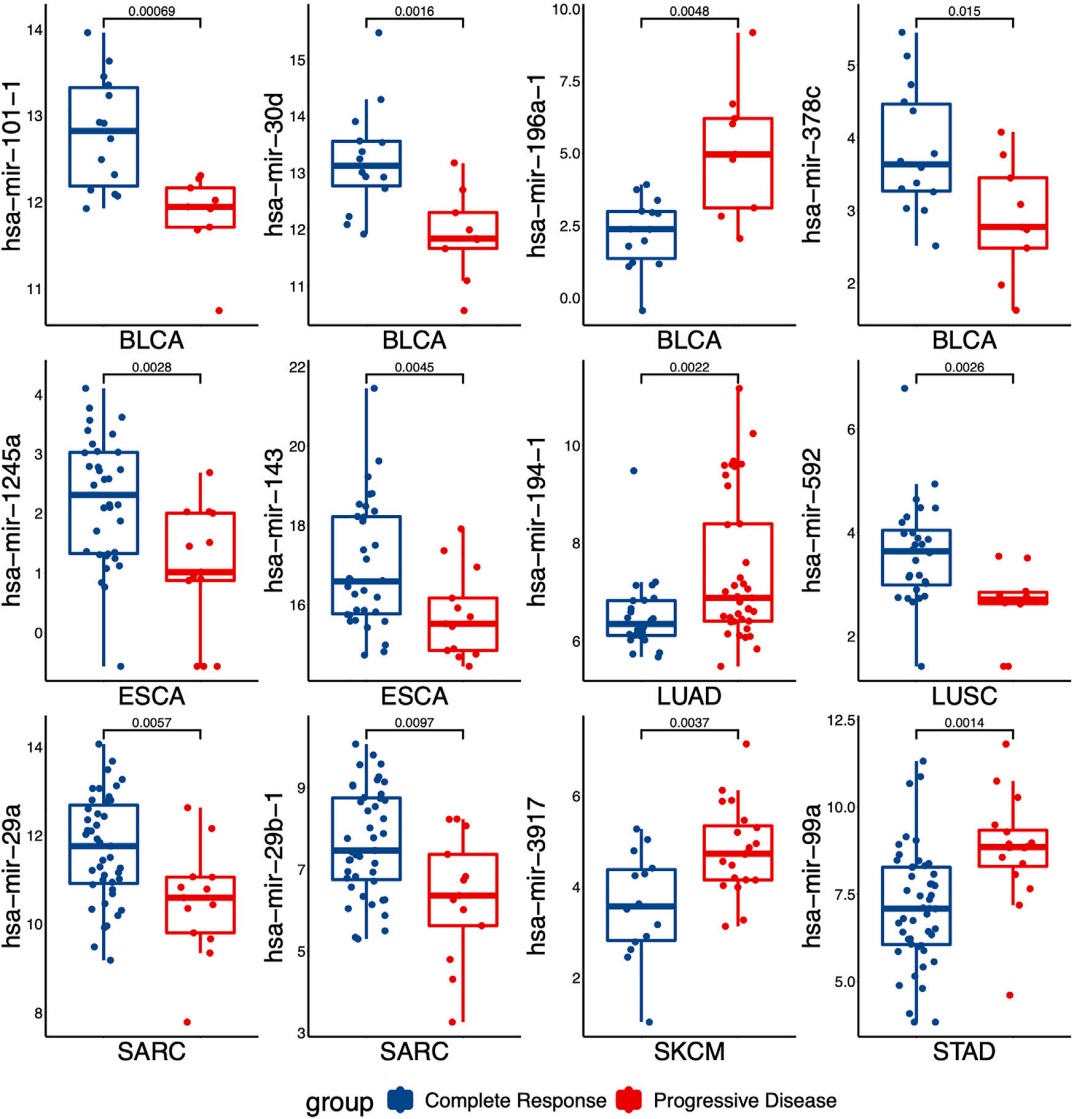
The levels of miRNAs expression that were differentially expressed between the responding (complete response) *versus* non-responding (progressive disease) tumors. *p* values were calculated by t-test.

**FIGURE 3:**
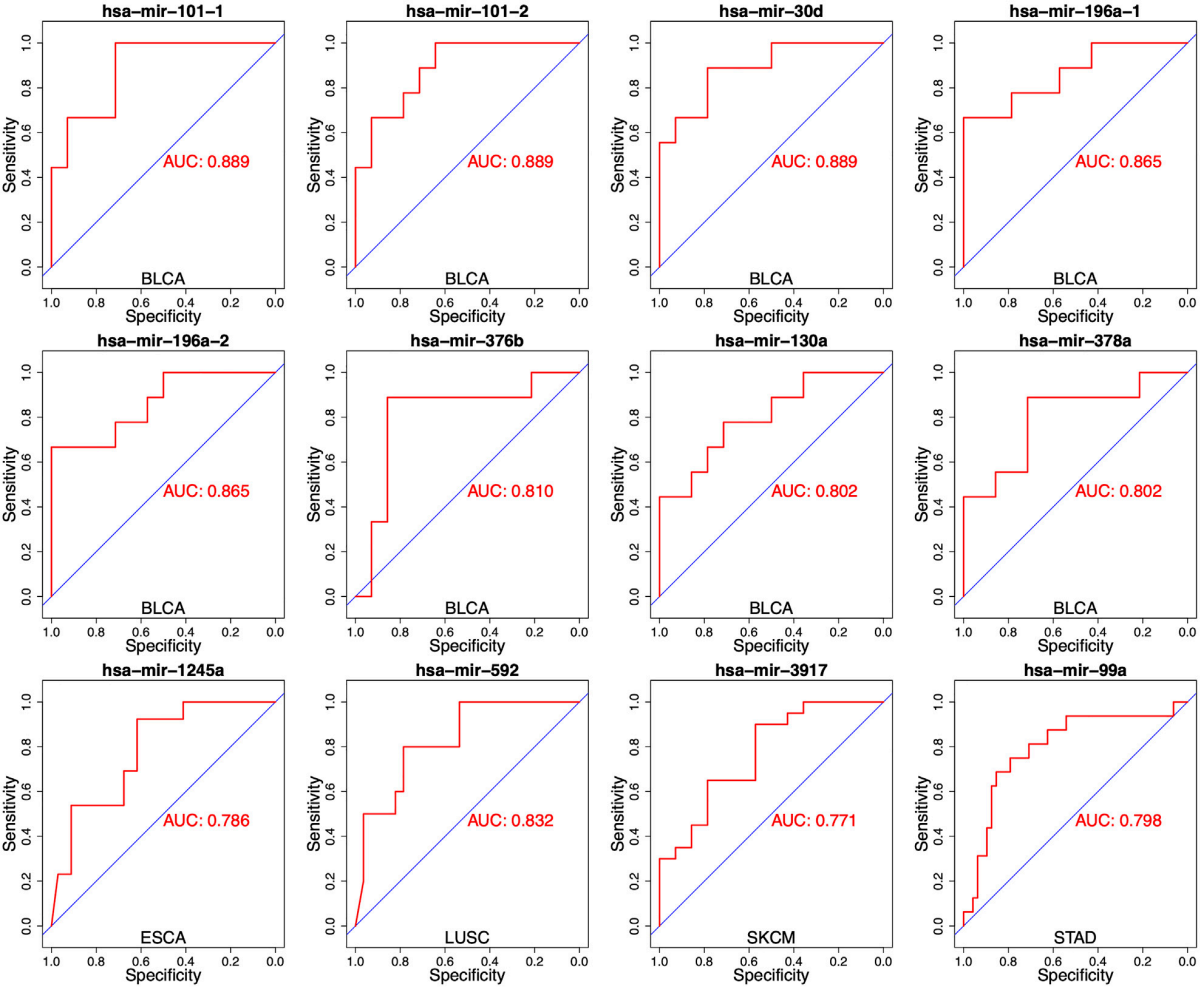
Receiver operating characteristics (ROC) analyses of the performance for the predictions for RT response using one single miRNA in different cancer types. The representative results were presented.

To evaluate the performance for distinguishing between S and R samples, we analyzed the specificity of each miRNA identified in DEMs list with the area under the curve (AUC). The expressed difference of individual miRNA for two groups was exhibited by boxplots. As shown in Figure 2, the expressed difference of individual miRNA was significant between RT responsive and non-responsive patients (*p* values <0.05, t-test). Particularly, miR-101-1 had the highest discrimination for the two groups in BLCA (*p* = 0.0007). Receiver operating characteristics (ROC) analyses were conducted to further assess the prediction performance of individual miRNA. As shown in Figure 3 several miRNAs had relatively high specificity for distinguish S and R samples, e.g., miR-101-1 (BLCA AUC = 0.889), miR-101-2 (BLCA, AUC = 0.889), miR-30d (BLCA, AUC = 0.889), miR-196a-1 (BLCA, AUC = 0.865), miR-196a-2 (BLCA, AUC = 0.865), and miR-592 (LUSC, AUC = 0.832). MiR-101 was known to be involved in the regulation of DNA damage repair and radiosensitivity. In our study, expression of miR-101 was up-regulated in RT responsive samples, hinting the high expression of miR-101 may be associated with radiosensitive phenotype. This is consistent with previous studies showing that up-regulation of miR-101 will enhance radiosensitivity (Yan et al., 2010). Our results implied that these miRNAs could be used as potential biomarkers for stratify patients treated with RT.

### 3.4 Using combinatorial model to predict RT response

**FIGURE 4:**
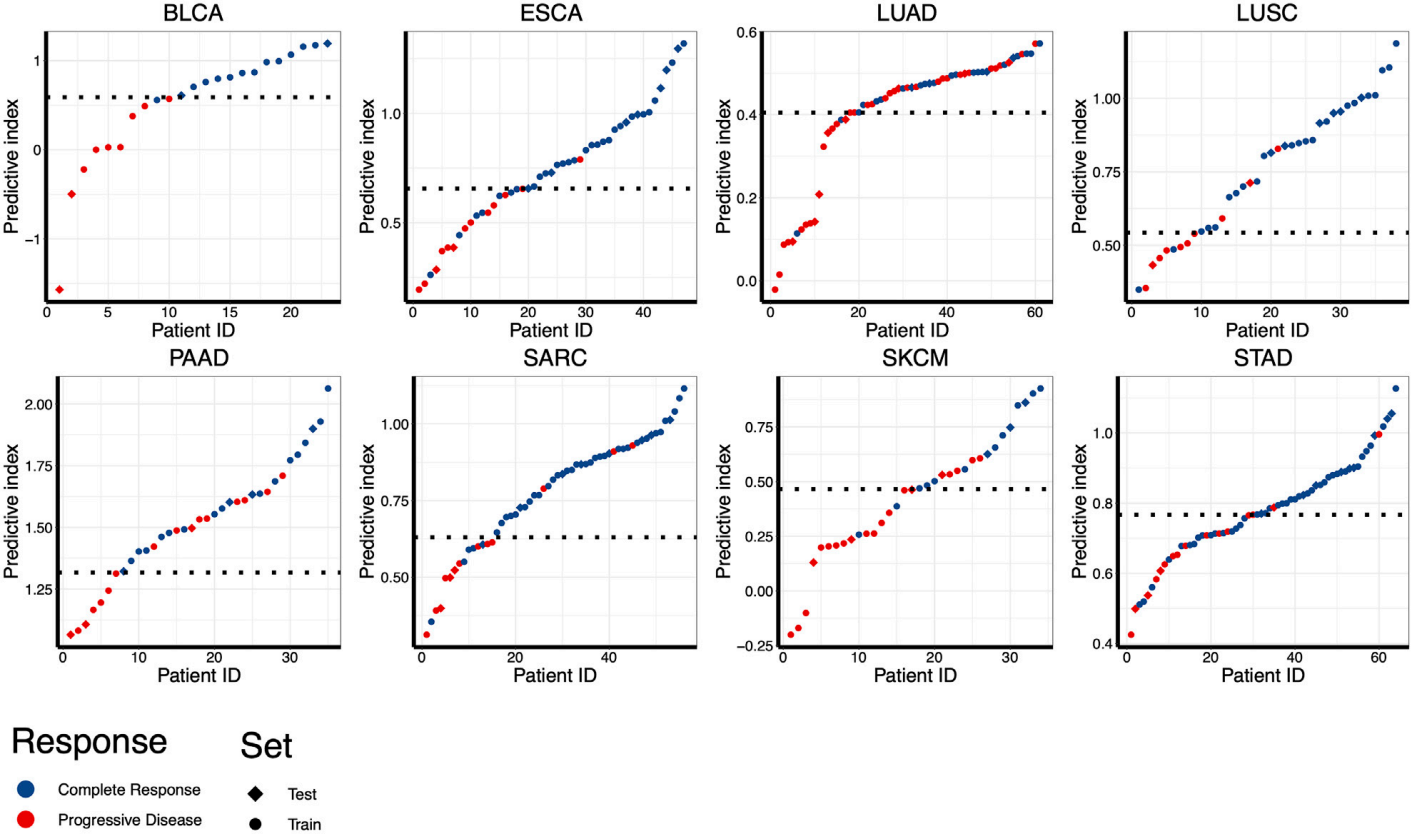
Assessing the performance for distinguishing RT responsive samples in eight different cancer types using combinatorial models. Predictive indexes were calculated to classify samples. Patients are shown in columns, and predictive index was showed in rows. The optimal cut-off value for distinguishing S and R samples was indicated by a dotted line. Samples of training set were indicated by circles, and samples of test set were indicated by diamonds.

**FIGURE 5:**
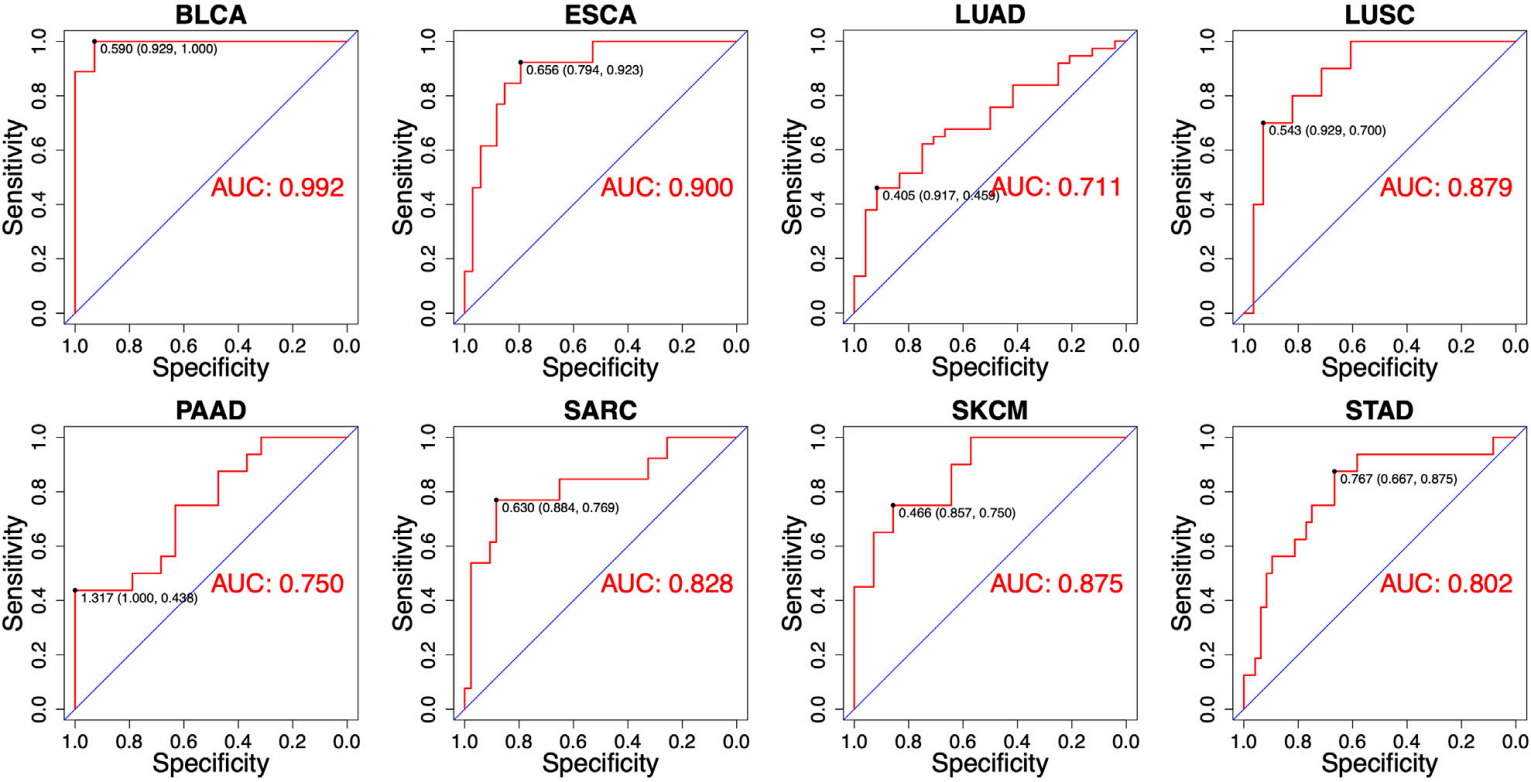
Receiver operating characteristics (ROC) analyses of the performance for the predictions for RT response using combinatorial models in eight different cancer types.

Despite the most of differentially expressed miRNAs could be used to distinguish patients according to RT response (e.g., AUC ranging from 0.667 to 0.786 in ESCA), further improvement on discriminatory ability is still needed. Therefore, we developed the combinatorial models based on a binary linear regression model. The combinatorial models for each cancer type consisted of 2-13 miRNAs (Supplementary Table S1). By applying this model, the best combination of multiple miRNAs for RT response prediction in each cancer type was identified (Supplementary Table S1). As showed in Figure 4, the optimal cut-off value for distinguishing S and R samples was indicated by a dotted line, and the predictive index of each sample was calculated for classification. The results showed that the most of S samples were above the threshold line, while the R samples were below the threshold line, implying that the most samples could be correctly classified in both training set and test set. Receiver operating characteristic (ROC) analysis revealed that the predictive reliability was significantly increased in all caner types by using these combinatorial models (Figure 5). For example, the AUC of single miRNA in LUSC was between 0.664 and 0.832. The AUC value was improved to 0.879 in of LUSC by applying the combinatorial models. Likewise, the combinatorial model improved the AUC to 0.900 in ESCA. These results indicated that the combinatorial model could significantly improv the AUC relative to the single miRNA.

### 3.5 Evaluation of survival rate with single miRNA signature

**FIGURE 6:**
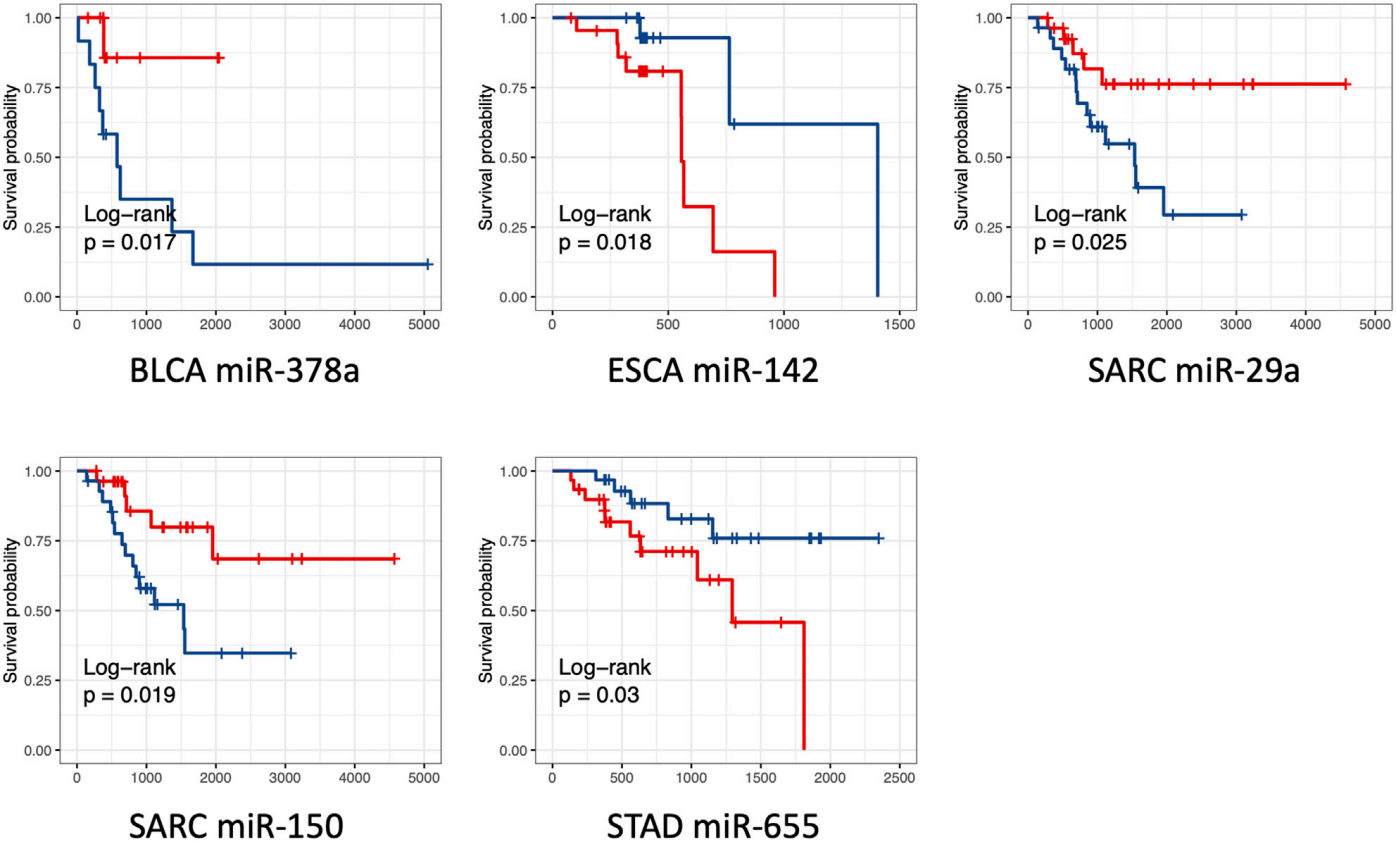
Kaplan Meier overall survival curves for patients with one single miRNA stratified by high *versus* low miRNA expression. Results from five miRNAs with low *p* values (*p* values <0.05) were shown. The high and low expression was indicated with red and blue color, respectively. *p* values were derived from log-rank test.

We also explored the potential link between these differentially expressed miRNAs and the overall survival of cancer patients. We found that the expression level of five miRNAs, including miR-378a (*p* = 0.017, in BLCA), miR-142 (*p* = 0.018, ESCA), miR-655 (*p* = 0.03, in STAD), miR-29a (*p* = 0.025, in SARC), and miR-150 (*p* = 0.019, SARC), were significantly associated with the overall survival time (Figure 6). Among them, miR-378a, miR-29a, and miR-150 tended to highly expressed in S group. The patients with high expression of these miRNAs showed a trend of better survival. In contrast, miR-142 (*p* = 0.018, ESCA) and miR-655 (*p* = 0.03, STAD) tended to be highly expressed in R group, and the worse survival was observed in these high expression samples. The results showed that only five miRNAs of 47 miRNAs were predictive for patient survival. It is not surprised since the differentially expressed miRNAs were identified based on the response to RT treatment rather than the overall survival of cancer patients.

## 4 Discussion

Currently, only few biomarkers have been evaluated for their radiotherapy-specific predictive value. Several research groups have reported that miRNAs were involved in regulation of radioresistance, hinting they have the potential serving as biomarkers for prediction of RT response. However, most of them was identified based on cellular experiments in previous studies. The evaluation based on clinical samples should be performed. In this study, we evaluated the predictive value of miRNA signatures for predicting RT response by using data of clinical samples. Comparison of miRNA expression of radioresistant and radiosensitive tumors led to the identification of 47 miRNAs. Most of them showed to be predictive for RT response (AUC ranging from 0.606 to 0.889). Some of them showed the high specificity for the prediction. For instance, miR-101, miR-30d, and miR-196s has AUC of 0.889, 0.889, and 0.865, respectively. To further improve the prediction performance, we developed a combinatorial model in each cancer type. In these models, the best combinations of multiple miRNAs could be obtained, which leads to an improved discriminatory power. The results showed that the most of AUC in each cancer type was greater than 0.8 when the combinatorial models were applied. Particular, the AUC value reach to 0.992 by applying the combinatorial model in BLCA. The prediction models and miRNAs identified here have the potential clinical application. As these combinatorial models contained 2-13 miRNAs, it is convenient to develop digital PCR and real-time qPCR based clinical test in routine practice (Hindson et al., 2013).

Identifying genetic clues to the molecular basis of radioresistance is a major challenge. Our study identified a series of miRNA that were differentially expressed between RT good responders and poor responders, providing useful clues for further functional assays to demonstrate a possible regulatory role in radioresistance. Among DEMs, several miRNAs were known to be involved in the regulation of DNA damage repair or radiosensitivity. For example, our results showed that miR-34a was upregulated in S group and was downregulated in R group in SARC. MiR-34a is one of the family members of miR-34 (Misso et al., 2014), and is a key regulator of tumor suppression. The overexpression of miR-34a makes cells sensitive to radiation by inhibiting several targets of DNA damage repair pathway (Lacombe and Zenhausern, 2017), such as Bcl-2 (Liu et al., 2011), LyGDI (Duan et al., 2013), Notch-1 (Kang et al., 2013), Rad51 (Cortez et al., 2015). MiR-29a is a member of the miR-29 family (Patel and Noureddine, 2012), and miR-29 is a tumor suppressor that can promote cell senescence and differentiation (Martinez et al., 2011; Chuang et al., 2022). The studies found that overexpression of miR-29a enhanced radiosensitivity, and promoted apoptosis in radiation resistant CaSki and c33a cells (Martinez et al., 2011). Overexpression of miR-29a induces a significant decrease in cell migration speed through K-ras/c-raf/p38 signal pathway, and may reduce metastasis of lung cancer (Chuang et al., 2022). In this study, we found that miR-29a in SARC tended to be highly expressed in S group. MiR-214 involved in radioresistance have been demonstrated in multiple cancer types (Zhang and Zhang, 2017; Hu et al., 2018; Li et al., 2019). In ovarian cancer, the expression level of miR-214 rises after ionizing radiation, which activates P13K/Akt pathway by targeting PTEN, resulting in the increased radioresistance of cell lines (Zhang and Zhang, 2017). In colorectal cancer, miR-214 is significantly down-regulated in cells after ionizing radiation, which resulting in the increased sensitivity of cells to radiation (Hu et al., 2018). MiR-214 is overexpressed in osteosarcoma tissues and is a negative regulator of phlda2, maintaining radioresistance of osteosarcoma cells to apoptosis (Li et al., 2019). We found that miR-214 tended to be highly expressed in the R group, implying it may play a role in radioresistance in BLCA. Besides, miR-101, miR-146a, miR-196a, miR-143, miR-222 (Shi et al., 2019), and mir-130a (Ha Thi et al., 2019), has been reported to be involved in the regulation of DNA damage repair or radiosensitivity in previous studies.

As several miRNAs previously reported to be associated with radiotherapy sensitivity (e.g., miR-34, miR-101, miR-29a, miR-214, miR-146a, miR-196a, miR-143, miR-222, and mir-130a), we hypothesized that the signatures identified in here would identify additional radiotherapy sensitivity-related miRNAs. Our gene function enrichment analysis showed the many miRNAs were involved in EMT pathway. EMT is associated with characteristics of cancer stem cells, including radioresistance and chemoresistance. Several miRNAs were reported to directly target multiple key components of EMT pathway. For example, the miR-200 family inhibits EMT and tumor metastasis, inhibits self-renewal of cancer stem cells, and enhances radiosensitivity of several types of cancer (Baumann et al., 2008). Overexpression of miR-200c regulates oxidative stress response genes and increases radiosensitivity of lung cancer cells (Magenta et al., 2011). Zheng et al. found that the down-regulation of miR-200c was related to radiation tolerance in esophageal squamous cell carcinoma (Zheng et al., 2017). Zhang et al. found miR-205 can suppress EMT by targeting the EMT-inducing transcription factor ZEB1 (Zhang et al., 2014). Function enrichment analysis indicated that 14 miRNAs, including miR-34a, miR-215, miR-221, miR-30d, miR-194-2, miR-let-7, miR-194-1, miR-129-1, miR-29b-2, miR-29b-1, miR-29a, miR-192, miR-150, and miR-143, were associated with EMT pathway in our study. This enrichment implied that these miRNAs might be involved in radioresistant phenotype through EMT pathway. Previous studies reported that miR192 regulated the EMT pathway (Khella et al., 2013). Song et al. confirmed that miR‐192 promoted EMT of gastric cancer, migration and invasion by targeting RB1 (Song et al., 2022). In additional, the previous study has demonstrated that miR-192 was significantly upregulated in cisplatin-resistant lung cancer cells, and miR-192 induced cisplatin resistance through activating the NF-κB pathway (Li et al., 2022). Moreover, miR-192 could influence 5-fluorouracil resistance (Boni et al., 2010). Cisplatin and 5-fluorouracil were genotoxic and their cytotoxic mode of action prominently involves the generation of DNA lesions followed by the activation of the DNA damage response and the induction of mitochondrial apoptosis. Zhai et al. found miR-143 suppressed epithelial–mesenchymal transition and inhibited tumor growth of breast cancer through down-regulation of ERK5 (Zhai et al., 2016). Up-regulating miR-143 enhances E-cadherin-mediated cell-cell adhesion ability, reduces mesenchymal markers, and decreases cell proliferation, migration, and invasion *in vitro* (Zhai et al., 2016). Yang et al. found that up-regulated miR-143 represses EMT in esophageal cancer cells (Yang et al., 2019). Our results showed that miR-143 was upregulated in S group and was downregulated in R group in esophageal cancer samples. We suggested that miR-143 might play a regulatory role in radiosensitivity through influencing EMT pathway in esophageal cancer. The further experiments will be required for function assay. We also noticed that miR-150 tended to be highly expressed in the RT response samples, implying it may play a role in radiosensitivity. Previous report showed that the expression of miR-150 decreased significantly in serum after irradiation in animal studies (Jia and Wang, 2022). Recently, miR-150-5p were confirmed to target ZEB1 and caused mRNA degradation, thus blocking EMT (Lu et al., 2017). Together, these multiple clues suggested that these miRNAs (miR-192, miR-143, and miR150) might serve as putative regulators of radiosensitivity through EMT pathway.

In conclusion, this work showed the miRNA signatures could serves as biomarkers to classify the RT response patients, and further investigations with larger numbers of patient samples are currently underway to validate the utility of using these biomarkers. Finally, additional work will be required to determine the role of these miRNAs in radioresistance of tumor.

## Data Availability

Publicly available datasets were analyzed in this study and are included in the article/Supplementary Material, further inquiries can be directed to the corresponding authors.
