# Supplementary material for: Improving the prediction for the response to radiotherapy of clinical tumor samples by using combinatorial model of MicroRNA expression
Source: Front Genet. 2022 Nov 22;13:1069112. doi: 10.3389/fgene.2022.1069112 (PMC9723130; doi:10.3389/fgene.2022.1069112)
Supplement: Supplementary file 1 [file DataSheet1.docx]

Supplementary Material

# Formulas of the Combinatorial Model of Eight Cancer types.

**BLCA**

Predictive Index = 0.21179 * miR-101-1 - 0.66759 * miR-101-2 + 0.63602 * miR-30d - 0.15080 * miR-196a-1 + 0.10870 * miR-196a-2 + 0.07286* miR-376b - 0.05863 * miR-151a + 0.18338 * miR-378c + 0.11997 * miR-214 + 0.35681 * miR-130a + 0.38744 * miR-378a + 0.28444 * miR-10a + 0.06630 * miR-944 - 12.66819

**ESCA**

Predictive Index = 0.13494 * miR-1245a + 0.05771 * miR-143 + 0.07763 * let-7e - 0.09456 * miR-142 - 0.29416

**LUAD**

Predictive Index = 0.01541 * miR-194-1 - 0.10266 * miR-194-2 - 0.05164 * miR-192 + 0.03610 * miR-215 + 1.36403

**LUSC**

Predictive Index = 0.10261 * miR-592 - 0.11165 * miR-937 + 0.05802 * miR-3653 - 0.02495 * miR-628 + 0.05833 * miR-106A + 0.34642

**PAAD**

Predictive Index = 0.503743 * miR-129-2 - 0.388578 * miR-129-1 - 0.007604 * miR-1224 + 0.960616

**SARC**

Predictive Index = -0.032834 * miR-29a + 0.406878 * miR-29b-1 - 0.340678 * miR-29b-2 + 0.020202 * miR-222 + 0.075057 * miR-34a - 0.145192 * miR-9-3 - 0.018247 * miR-9-1 + 0.151409 * miR-9-2 - 0.050383 * miR-582 - 0.017762 * miR-146a - 0.021003 * miR-221 - 0.003514 * miR-150 + 0.808718

**SKCM**

Predictive Index = -0.128413 * miR-3917 - 0.140905 * miR-33a - 0.080170 * miR-3130-1 - 0.001194 * miR-3614 + 2.101791

**STAD**

Predictive Index = -0.06247 * miR-99a - 0.05225 * miR-655 + 1.32159
